# Metagenomic Insights into the Phylogenetic and Metabolic Diversity of the Prokaryotic Community Dwelling in Hypersaline Soils from the Odiel Saltmarshes (SW Spain)

**DOI:** 10.3390/genes9030152

**Published:** 2018-03-08

**Authors:** Blanca Vera-Gargallo, Antonio Ventosa

**Affiliations:** Department of Microbiology and Parasitology, Faculty of Pharmacy, University of Sevilla, 41012 Sevilla, Spain; vera@us.es

**Keywords:** metagenomics, saline soils, hypersaline environments, metagenome assembled genomes

## Abstract

Hypersaline environments encompass aquatic and terrestrial habitats. While only a limited number of studies on the microbial diversity of saline soils have been carried out, hypersaline lakes and marine salterns have been thoroughly investigated, resulting in an aquatic-biased knowledge about life in hypersaline environments. To improve our understanding of the assemblage of microbes thriving in saline soils, we assessed the phylogenetic diversity and metabolic potential of the prokaryotic community of two hypersaline soils (with electrical conductivities of ~24 and 55 dS/m) from the Odiel saltmarshes (Spain) by metagenomics. Comparative analysis of these soil databases with available datasets from salterns ponds allowed further identification of unique and shared traits of microbial communities dwelling in these habitats. Saline soils harbored a more diverse prokaryotic community and, in contrast to their aquatic counterparts, contained sequences related to both known halophiles and groups without known halophilic or halotolerant representatives, which reflects the physical heterogeneity of the soil matrix. Our results suggest that *Haloquadratum* and certain Balneolaeota members may preferentially thrive in aquatic or terrestrial habitats, respectively, while haloarchaea, nanohaloarchaea and *Salinibacter* may be similarly adapted to both environments. We reconstructed 4 draft genomes related to Bacteroidetes, Balneolaeota and Halobacteria and appraised their metabolism, osmoadaptation strategies and ecology. This study greatly improves the current understanding of saline soils microbiota.

## 1. Introduction

Extreme environments are habitats in which biodiversity is severely restrained by one or several physico-chemical factors [1]. Hypersaline environments comprise examples of such habitats in which the main environmental factor limiting life is their high salt concentration. The harshness of hypersaline environments is often increased by concurrent low concentrations of dissolved oxygen and nutrients, extreme pH values and high temperatures, pressures, and the presence of toxic compounds. These environments include saline lakes, marine salterns, salted foods, deep-sea brine pools and saline soils and sediments [2]. While extensive studies have been performed on aquatic hypersaline habitats [2,3,4], other saline environments such as soil systems have been much less thoroughly investigated [5].

With an estimated extension of 397 million hectares, salt-affected soils comprise more than 3% of the world’s land area. Soil salinity negatively impacts agricultural production and leads to changes in microbial community composition and activity [6]. Microorganisms are central to biogeochemical cycles and thus play essential roles in the provision of soil services [7]. Despite the economic, environmental, and nutritional consequences of the increasing rate of soil salinization, microbial biodiversity studies of saline soils are scarce [8]. Furthermore, the few available studies focused on either bacteria [9,10,11,12,13] or archaea [14,15,16,17], but rarely on both domains [5,18,19,20], which has led to a partial and incomplete view of the phylogenetic diversity and potential microbial processes carried out in these soils. Overall, our current aquatic-biased knowledge about halophilic microorganisms and their environments, and the limited data available from saline soil studies, impair our ability to make knowledge-based predictions of the effects of the increasing salinization of soils as well as to direct management and rehabilitation plans.

Metagenomic techniques enable the investigation of biodiversity avoiding the great plate count anomaly and PCR biases [21]. Recent research into the prokaryotic diversity and ecology of aquatic hypersaline environments addressed by shotgun metagenomics revealed a change in biodiversity, abundance of specific taxa and osmoadaptation mechanisms along a salinity gradient [22,23,24,25], uncovered the presence of new phylogenetic groups [22,25,26] and even fostered the isolation of previously untapped taxa in pure culture [27]. On the other hand, metagenomic studies assessing prokaryotic biodiversity of saline soils have provided a first glimpse into the structure of their microbial community but have not allowed the recovery of metagenome assembled genomes (MAGs) that would shed light on the physiology and ecology of yet uncultured abundant taxa present in these environments. 

Here we present a snapshot of the phylogenetic and metabolic diversity of the prokaryotic microbiota dwelling in saline soils located in the Odiel saltmarshes (Southwest Spain) obtained by analyzing two 454 shotgun metagenomes and the reconstructed draft genomes from them. Studies of the zonation, diversity, and restoration potential of plants adapted to the conditions in this brackish and metal-contaminated estuary have been carried out in this area [28,29,30], but microbiota from this habitat has been largely disregarded until now. The integration of our results with those from extensively studied aquatic systems and the limited available biodiversity studies on saline soils has allowed to evaluate the biases of the current knowledge about halophiles and their ecology. 

## 2. Materials and Methods 

### 2.1. Sampling Site and Sample Collection

The Odiel saltmarshes, an area designated as Biosphere Reserve by the UNESCO and Wetland of International Importance (Ramsar Convention) [31], is situated in the estuary of the Odiel and Tinto rivers (Huelva, Southwest Spain). With a Mediterranean climate, the location has a dry season during the summer and receives frequent rainfall in autumn and winter. Mean annual precipitation is 506 mm. Mean, maximum and minimum air temperatures reported in the area are 18.3, 31.6 and 7.7 °C, respectively. The average tidal range is 2.10 m with high tides rising to 4 m during equinox [29,32]. Tidal cycles create distinct habitats within marsh creeks: saturated (low), intermittently flooded (medium), and unsaturated (high) saltmarsh zones. Plants most frequently found on the vegetated areas comprise *Spartina densiflora, Salicornia ramossisima* and *Sarcocornia* [32]. Draining the Iberian Pyrite Belt, the Odiel and Tinto rivers transport a variety of metals through the saltmarshes, which are also affected by industrial effluents from factories located nearby [33,34].

The two soil samples were collected from the same bare high saltmarsh area (37°12’26” N, 6°57’58” W) in October 2013 and November 2014, and were designated SMO1 and SMO2, respectively. Approximately 200 g of soil was collected in Whirl-Pak bags during each period and kept on ice during their transport to the laboratory. 

### 2.2. Physico-Chemical Characterization of Soils

The two soil samples were submitted to Innoagral (Grupo Hespérides Biotech S.L., Sevilla, Spain) for physico-chemical analysis. Texture was evaluated by the Bouyoucos method and United States Department of Agriculture (USDA) textural classification system was used for determining textural class [35,36]. Water content was measured gravimetrically. pH was determined electrochemically in 1:2.5 soil to water extracts, and salinity measurements were carried out by conductivity in 1:5 soil to water extracts. Carbon was measured according to Walkley-Black [37]. Kjeldhal method was employed for nitrogen content determinations [38]. Ammonia, sulfate, phosphorus, nitrate, and nitrite contents were determined in 1:5 extracts by UV-visible spectroscopy. Volumetry was used for measuring chloride concentrations. Atomic absorption spectroscopy was employed to determine salinity-related ions content and metals concentration in these soils.

### 2.3. DNA Extraction and Sequencing

Total DNA was extracted from 10 g of soil from each sample using FastDNA SPIN Kit for Soil (MP Biomedicals, Santa Ana, CA, USA) following the manufacturer’s instructions. Further purification of the DNA was carried out with phenol/chloroform method [39]. The two metagenomic databases were obtained using 454 pyrosequencing technology available at the Biology Service in Centro de Investigación, Tecnología e Innovación de la Universidad de Sevilla (CITIUS), Sevilla, Spain.

### 2.4. Bioinformatics Analysis of the Databases

Metagenomic reads were quality checked and filtered using FastQC v0.10.1 [40], QUAST v2.3 [41] and Prinseq v0.20.3 [42]. GC content and isoelectric point of predicted proteins were computed with the EMBOSS package v.6.5.7.0 [43], while amino acids frequency was determined with a custom Perl script. The 16S ribosomal RNA (rRNA) genes were identified in reads by BLASTn comparison against RDP database v11.4 [44]. Results with a hit alignment longer than 100 bp and an e-value lower than 1 × 10^−5^ were retained. Sequences with ≥80% and ≥95% identity to their matches were considered for phylum and genus level characterization, respectively.

Reads were assembled using Newbler v2.9 [45] and assembly quality checked with QUAST v2.3. Contigs longer than 1 kb were considered for subsequent analysis. Taxonomic annotation of reads and contigs was performed using the lowest common ancestor (LCA) algorithm in MEGAN v6.5.10 [46] with default parameters and non-redundant (nr) database from September 2017. When possible, new phyla incorrectly placed in nr database were manually curated. Functional annotation of reads and contigs was carried out using COGNIZER v0.9b [47]. Rarefaction curves were computed with QIIME pipeline v1.9.1 [48]. 

Binning was performed with MetaBAT v0.26.3 [49]. CheckM v1.0.5 [50] was then used for assessment of completion and contamination of the extracted genomes or bins. Further refinement of bins was carried out with VizBin v0.9 [51]. Their taxonomic affiliation was determined by constructing a genomic tree with FastTree v2.1.3 [52] of the concatenation of PhyloSift v.1.0.1 [53] set of conserved genes from the extracted bins and reference genomes obtained from National Center for Biotechnology Information (NCBI) Refseq database. Average nucleotide identity (ANI) was calculated following the method OrthoANIu described by Yoon et al. [54]. 

Genomes were annotated in RAST server v2.0 using RASTtk annotation scheme [55]. Fragment recruitment analysis was performed by mapping metagenomic reads from diverse hypersaline environments with a minimum of 70% identity to the Balneolaeota-related extracted genome. The percentage of reads recruited was normalize by database size.

In this study, several publicly available metagenomic databases from hypersaline aquatic habitats were also analyzed for comparison purposes. Those included datasets from saltern ponds with different salinities (13% to 37%) located in Santa Pola (Alicante, SE Spain) [22,23] and Isla Cristina (Huelva, SW Spain) [56], all of them described in Table 1. Metagenomic datasets used in recruitment analysis encompass a marine database of the deep chlorophyll maximum from the Mediterranean Sea (DCM) [57], a dataset from a crystallizer pond with 34% salinity from Cáhuil Lagoon (Chile) [58], the datasets obtained from Guerrero Negro solar saltern (Baja California Sur, Mexico) [24] and the metagenomes from soils located in the Great Rann of Kutch (Gujarat, India) [19]. Metagenomic datasets from the same locations were combined in our analysis.

### 2.5. Data Availability

The raw sequence reads of the two saline soil metagenomic databases and the 4 reconstructed draft genomes have been deposited to DDBJ/ENA/GenBank under the project PRJNA318875 [59].

## 3. Results and Discussion

### 3.1. Environmental Data

The physico-chemical properties of the studied saline soils are summarized in Table S1 in the Supplementary Material. With an electrical conductivity of the 1:5 soil to water extract (EC_1:5_) of ~24 and 55 dS/cm, the studied soils could be classified as saline according to the several classifications available [60,61,62]. The water content of the soils at the sampling moments was relatively low. Although sodium was the dominant cation in both samples, the sampling area showed to be dynamic with respect to the contributions of the different anions and cations to salinity from one year to the other. We observed a higher salinity, pH and organic matter content in the sample retrieved in November 2014. Metal content, that we included in the analysis due to evidences of high metal contamination of sediments from the Odiel saltmarshes [33,34], did not surpass the legal limits to consider the studied soils as metal-contaminated ones. These soils were classified as sandy loam according to USDA textural classification system. Organic carbon content was in the range of the values reported for saline soils of La Sal del Rey (Edinburg, TX, USA) [18] and Sicily (Italy) [10], but lower than those found in saline soils of Qarhan Salt Lake (China) [5]. Nitrogen contents of the studied mineral soils are similar to those of the study of Hollister et al. [18] and Pandit et al. [19]. 

Trying to assess the harshness of the environment by comparing salinities across hypersaline habitats is problematic. Contrary to measurements in hypersaline aquatic systems, soil salinity, as commonly evaluated (in saturated paste, 1:2, 1:5 or 1:10 soil to water ratio), has been found to be a poor estimate of the osmotic and ionic stress suffered by the microbiota inhabiting these environments, especially the driest ones, both due to the variability at the smaller scales and other factors influencing osmotic stress in soils (e.g., texture) [63]. Furthermore, the use of different soil to water extractions ratios in the measurement of soil salinity makes it challenging to make comparisons among different saline soil studies, as conversions are not simple. Other measurements proposed to circumvent the scale and matric effect problems [63,64] are not widely employed. 

Thus, we did not venture to compare soil salinity levels with those from hypersaline aquatic systems previously reported, as it could lead to an inaccurate estimation of the osmotic stress suffered by microorganisms in these saline soils. However, with that in mind, we attempted to situate our study in the context of the available literature about saline soils microbiota, using proposed conversions when necessary [65]. We found that the soils considered in our study were in the range of the saline soils studied by Xie et al. from Qarhan Salt Lake (China) [5] and highest salinity soils addressed by Hollister et al. [18], Canfora et al. [10], Pandit et al. [19] and Navarro-Noya et al. [14] from La Sal del Rey (Edinburg, TX, USA), Sicily, the Great Rann of Kutch desert (Gujarat, India) and the former Lake Texcoco (Mexico), respectively.

### 3.2. General Characteristics and Halophilic Traits of the Databases

Table 1 displays the general features of the studied saline soil metagenomic datasets, SMO1 and SMO2, as well as those of the publicly available 454 metagenomic databases from hypersaline aquatic habitats chosen as reference. Since sequencing technology and analysis workflow has been shown to greatly affect comparability of metagenomic studies results [66], publicly available 454 metagenomic databases from salterns were analyzed in parallel to the newly sequenced soil databases. It is worth noting the relatively large metagenomic reads obtained from these saline soil samples (with average read length of 628 and 629 bp), and the low proportion of them that could be assembled into contigs, as compared to aquatic datasets.

Several genomic features, such as an acidic proteome and a high GC content, have been proposed as physiological adaptations of microorganisms to hypersaline habitats [67]. Hence, these traits have been used in previous studies to characterize metagenomic datasets from aquatic saline environments and to get the first insights into the microbial community living in them [22,23,56,68].

**Figure 1:**
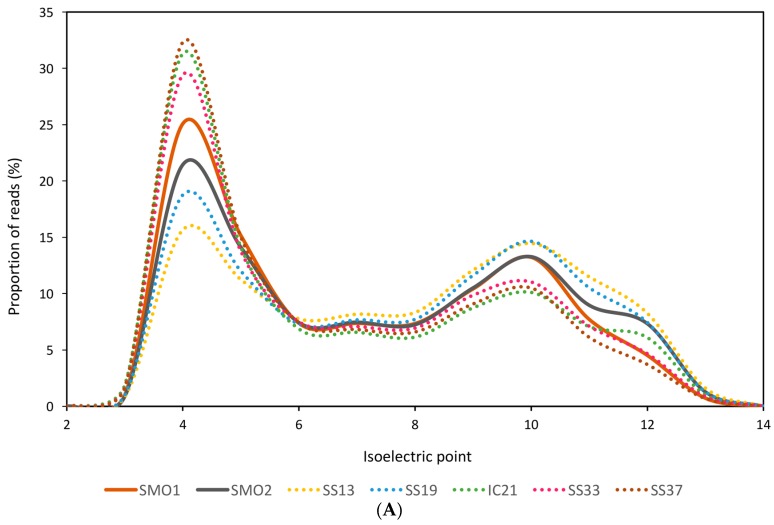

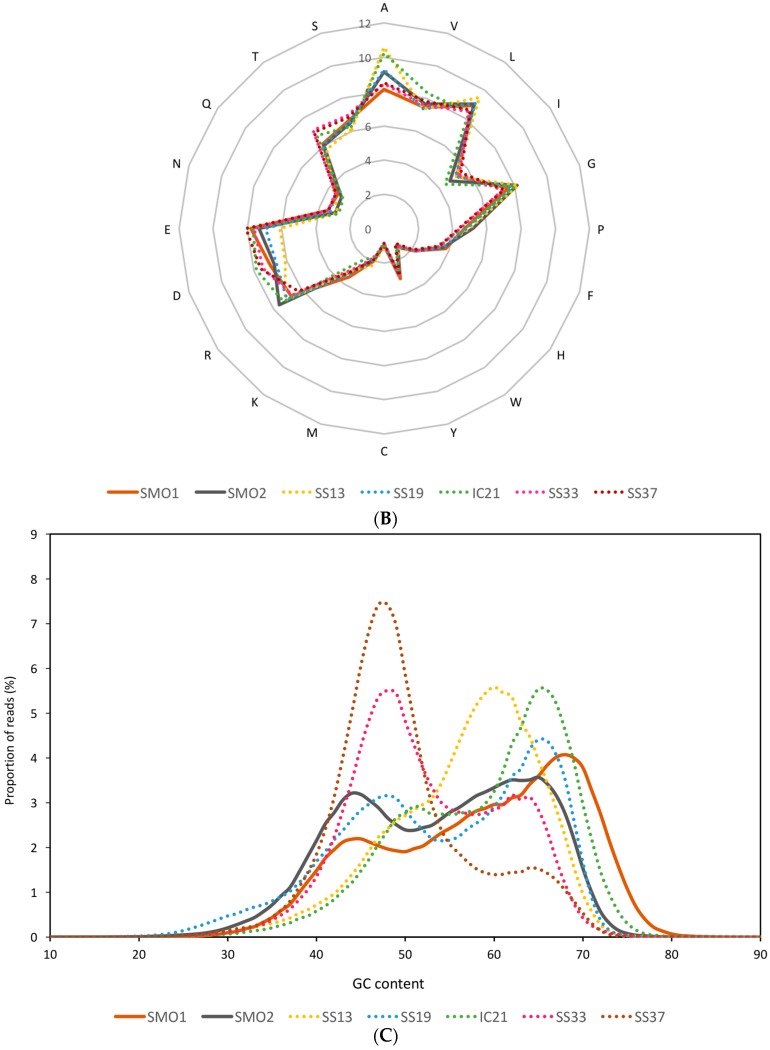
General characteristics of the saline soil metagenomic datasets, as compared to saltern metagenomic databases: (**A**) Isoelectric point of predicted proteins computed for each translated read and shown as a percentage of the total reads in the dataset, in intervals of bin width 2; (**B**) Frequency of use of amino acids of the predicted proteins; (**C**) GC content of the reads. Databases used are described in Table 1.

Figure 1A depicts the predominantly acidic isoelectric point of the predicted proteins from soils and salterns databases considered. The soil databases analyzed in this study followed the trend of amino acids use in the hypersaline aquatic systems, being acidic residues more frequently employed and arginine utilized as the preferred basic amino acid (Figure 1B) [56].

Here we show that the GC content of the reads from the saline soils studied followed a bimodal distribution similar to those from salterns (Figure 1C). Most extreme halophiles, such as members of the class Halobacteria (Euryarchaeota) and the bacterial genus *Salinibacter*, are characterized by a high genomic GC content, which has been hypothesized to help them protect against thymidine dimers formation caused by the high solar radiation that most hypersaline environments are subjected to [67,69,70]. An exception to this rule is the square haloarchaeon *Haloquadratum walsbyi*, whose GC content is 47.9% [71]. While the two GC peaks of saltern ponds were clearly defined and related to the GC contents of the abundant taxa belonging to the genus *Haloquadratum* and other members of the class Halobacteria [22,23,56], the bimodal distribution of the GC content of sequences from saline soil databases was vaguer with wider peaks. Together with the broader range of GC contents exhibited by the microbiota from soil databases, these data suggest that the community inhabiting saline soils is more diverse than the one in saltern ponds, and that there is less predominance of particular groups. In saline soils, the low GC content peak does not seem to relate to *Haloquadratum walsbyi* genomic GC content, but may accommodate other halotolerant and halophilic taxa of low GC such as the candidate group nanohaloarchaea, and members of the phyla Bacteroidetes, Balneolaeota or *Firmicutes* [22,25,26,72]. On the other hand, the high GC content peak may correspond to the halophilic strains within Euryarchaeota, Rhodothermaeota or Actinobacteria [22,72,73,74]. Despite the high salt content of the studied soils, and because of the high heterogeneity of soil structure, a fraction of the saline soils metagenomic sequences may also correspond to non-halophiles. 

### 3.3. Diversity Estimates

**Figure 2:**
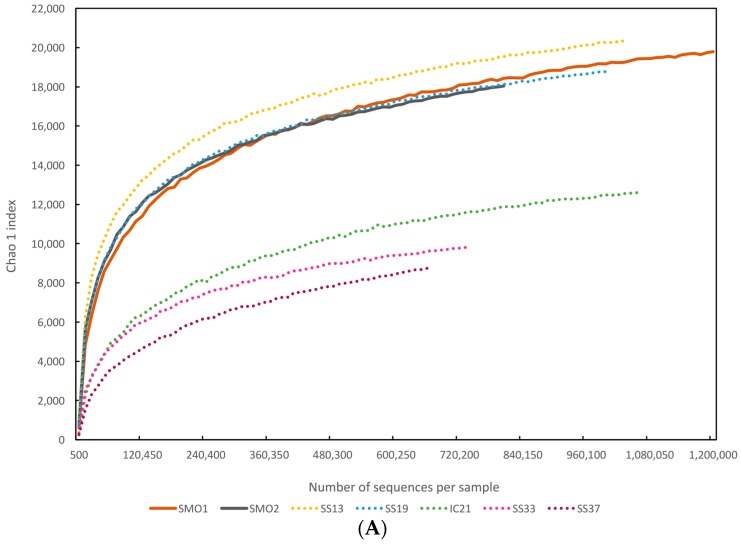

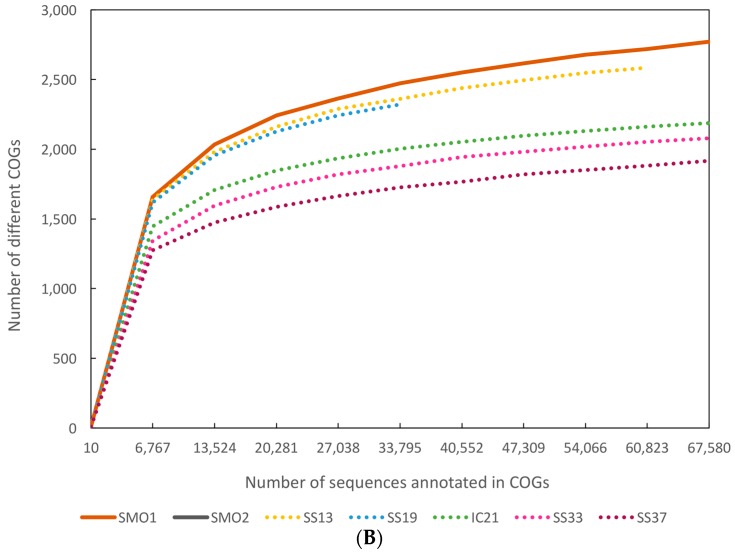
Rarefaction curves-based of the phylogenetic and metabolic prokaryotic diversity of the studied databases; (**A**) Rarefaction plot of the phylogenetic diversity, using Chao 1 index as alpha diversity metric; (**B**) Rarefaction plot of the functional diversity. Databases used are described in Table 1. COGs: Clusters of orthologous groups.

To evaluate the extent of diversity captured in this study we performed rarefaction analysis based on phylogenetic and functional annotation of reads (Figure 2A,B). Rarefaction curves showed that we captured a good proportion of the microbial diversity in these saline soils (Figure 2). Microbial diversity (evaluated by Chao 1 richness estimator) of the saline soil databases was in the range of the most diverse and less saline salterns datasets, SS13 and SS19 (Figure 2A). As for functional diversity, rarefaction plot based on clusters of orthologous groups (COGs) annotated in each dataset is depicted in Figure 2B. Saline soil metagenomes comprised a higher functional diversity versus salterns datasets. These results agree with studies describing soil environments as the most diverse environments on earth [75], presumably due to both a higher heterogeneity of the soil structure, which promotes the existence of micro-niches with different environmental conditions, and higher disturbance rates [16,64,76]. In particular, the considered soils were unsaturated, a situation in which connection between niches is mostly impaired and thus, spatial isolation of groups of microorganisms occurs [64,77]. 

### 3.4. Microbial Community Composition

To confidently annotate taxa and functions, we assembled reads into contigs. In the case of the more diverse saline soil databases, we co-assembled the two metagenomes obtained and use tetranucleotide frequency and differential coverage-based binning, which has been shown to improve taxonomic binning and recovery of draft genomes from complex metagenomes [78,79]. Despite co-assembly and a longer average length of soil databases reads, only a 26.7% of the sequences could be assembled into contigs (Table 1). Our previous studies on the crystallizer pond of a saltern from Santa Pola showed that the prokaryotic community was largely dominated by the square archaeon *Haloquadratum walsbyi* and, in a lower proportion, by the extremely halophilic bacterium *Salinibacter* [22]. This reduced diversity permitted the assembly of reads in contigs up to ~70%. In comparison, hypersaline saline soils studied here showed a much higher prokaryotic diversity which justifies the lower proportion of assembled reads achieved. Once more, this emphasizes the high taxonomic richness and low dominance of the studied saline soils, as compared to salterns databases.

**Figure 3:**
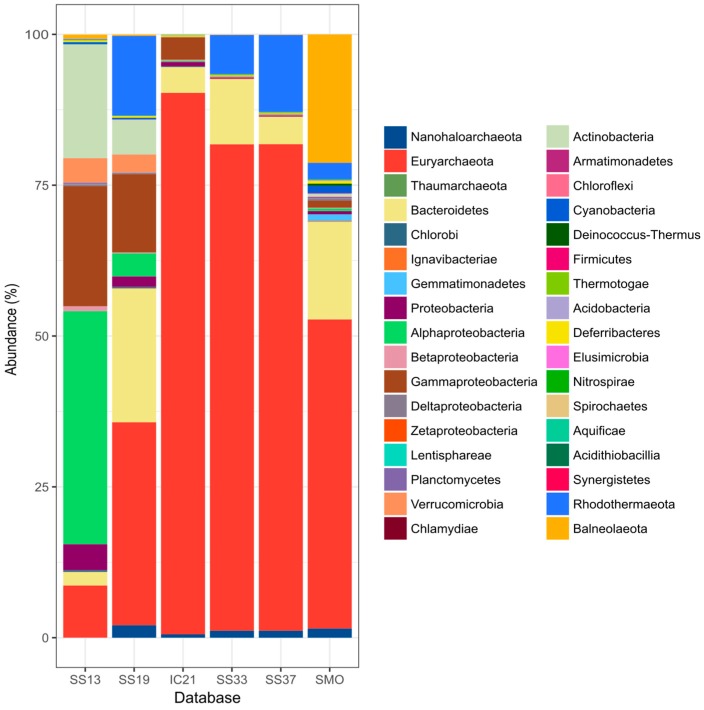
Microbial diversity at the phylum level represented in contigs longer than 1 kb, annotated with MEGAN v6.5.10 LCA method (see Materials and Methods section). Salterns databases used are described in Table 1. SMO refers to the contigs resulting from the co-assembly of the two saline soil databases, SMO1 and SMO2.

Taxonomic affiliation of contigs is shown in Figure 3. The archaeal fraction in saline soils comprised roughly half of the microbial community. This ratio of archaea to bacteria is consistent with that reported for other saline soils with the highest salinities [19]. The class Halobacteria, represented by obligate and extreme halophiles, was the dominant archaeal taxon, as is also the case in most of the hypersaline habitats studied to date [2,3,4]. Of notice is the presence of contigs related to Nanohaloarchaeota. This candidate phylum was discovered in an intermediate salinity habitat and, although it has since been observed in a wider range of aquatic saline environments [22,25,80,81,82,83,84,85,86,87,88], it has not previously been detected in soils. A small proportion of sequences related to Thaumarchaeota, a phylum of ammonia-oxidizing organisms currently not known to harbor halophiles [89], was also detected in the saline soil metagenomic datasets. Despite Thaumarchaeota having been found to be the most abundant group of archaea in aquatic and terrestrial habitats [90] only a minority of studies have reported its presence in saline soils [5,14,90]. On the other hand, we did not detect the presence of archaeal methanogens or members of the phylum Crenarchaeota, which have been identified in saline soils before [14,16,19].

The bacterial community from the saline soil databases studied, with 26 different phyla represented, was more diverse than that described for different salinity ponds of salterns [22,23,56]. Similar to the archaeal population, bacterial fraction comprised phyla containing well-known halophiles, such as Actinobacteria, Bacteroidetes, Cyanobacteria, Firmicutes, Proteobacteria, Spirochaetes, and Thermotogae, as well as groups that do not encompass described halophiles [89]. This may be due to the halophilic properties of not yet described strains and/or the existence of micro-niches in soil with a wide variety of salinity conditions. In fact, other studies have detected non-halophiles in saline soils, comprising up to 20% of the community in some cases [10,14,20].

Table 2 shows the taxonomic affiliation of sequences related to 16S rRNA genes found in SMO1 and SMO2 databases. Surprisingly, we did not detect any sequence related to the genus *Haloquadratum*, the most abundant archaeal taxon at high salinity saltern ponds [22,23,56,91], which confirms that this organism is not an abundant dweller of these soils, as suggested before by GC content analysis (Section 3.2). This result agrees with previous surveys of archaea in saline soils, except for one study which detected sequences related to *Haloquadratum* in the desert of the Great Rann of Kutch (Gujarat, India) [19]. In the studied soils, other haloarchaeal genera such as *Haloarcula*, *Halorubrum*, *Salinigranum*, *Halolamina* and *Halobellus*, as well as different members belonging to the bacterial phyla Bacteroidetes, Rhodothermaeota and Balneolaeota were the dominant taxa (Table 2). All those genera have been found in aquatic and terrestrial hypersaline habitats before [92,93]. Thus, while some non-salinity-related taxa have been detected in the studied soils, the prevailing genera contained known halotolerant or halophilic species.

Our data revealed that sequences related to members of the genus *Salinibacter*, which share habitat with *Haloquadratum walsbyi* in highly saline aquatic environments, comprised more than 4% of the sequences in SMO1 database, but this genus was not among the most abundant taxa in SMO2. Thus, even when *Salinibacter* can thrive in the studied saline soils, the variation in physico-chemical parameters suffered by these soils from October 2013 to November 2014 did not foster its growth. Methanogenic halophiles, halophilic sulfate reducers and the phylogenetically coherent group of anaerobic halophiles Halanaerobiales (Firmicutes), did not seem to be members of the studied microbial community, pointing towards limited sustained anaerobic conditions in these soils and salterns. 

We did not observe a clear dominance of any particular genus (Table 2), which reflects the diverse and even community present in the studied soils. It is worth noting the high proportion of genera constituting less than 10% of the reads of each metagenome, grouped in “Others” category. In the case of SMO2, this category comprised more than 20% of the population. Hence, species richness was higher in SMO1, which also had higher species dominance, being phylogroups more evenly distributed in SMO2. Species richness of both soil databases were similar to that of saltern ponds with intermediate salinity (13% and 19%) [23]. 

Notably, in both soil databases more than 6% of the 16S rRNA-related reads corresponded to sequences from the class Halobacteria not classified at the genus level. Sequences related to unclassified Gammaproteobacteria also constituted more than 3% of the reads related to 16S rRNA genes in the soil databases. These results are in accord with previous studies that showed that the microbial community in saline soils contained a considerable proportion of sequences distantly related to those in public databases [16,18,93].

Overall, most of the genera detected in these soils comprise halophilic representatives commonly found in aquatic habitats. However, some well-known halophiles abundant in aquatic systems are missing in these saline soils. Bacterial diversity is higher than in aquatic ecosystems and some groups without known halophilic representatives are also present in soils, albeit in low proportions, which suggests that the specific properties of terrestrial and aquatic habitats may be key in determining the presence and abundance of highly specialized microorganisms and those others variably adapted to salinity.

### 3.5. Functional Diversity

**Figure 4:**
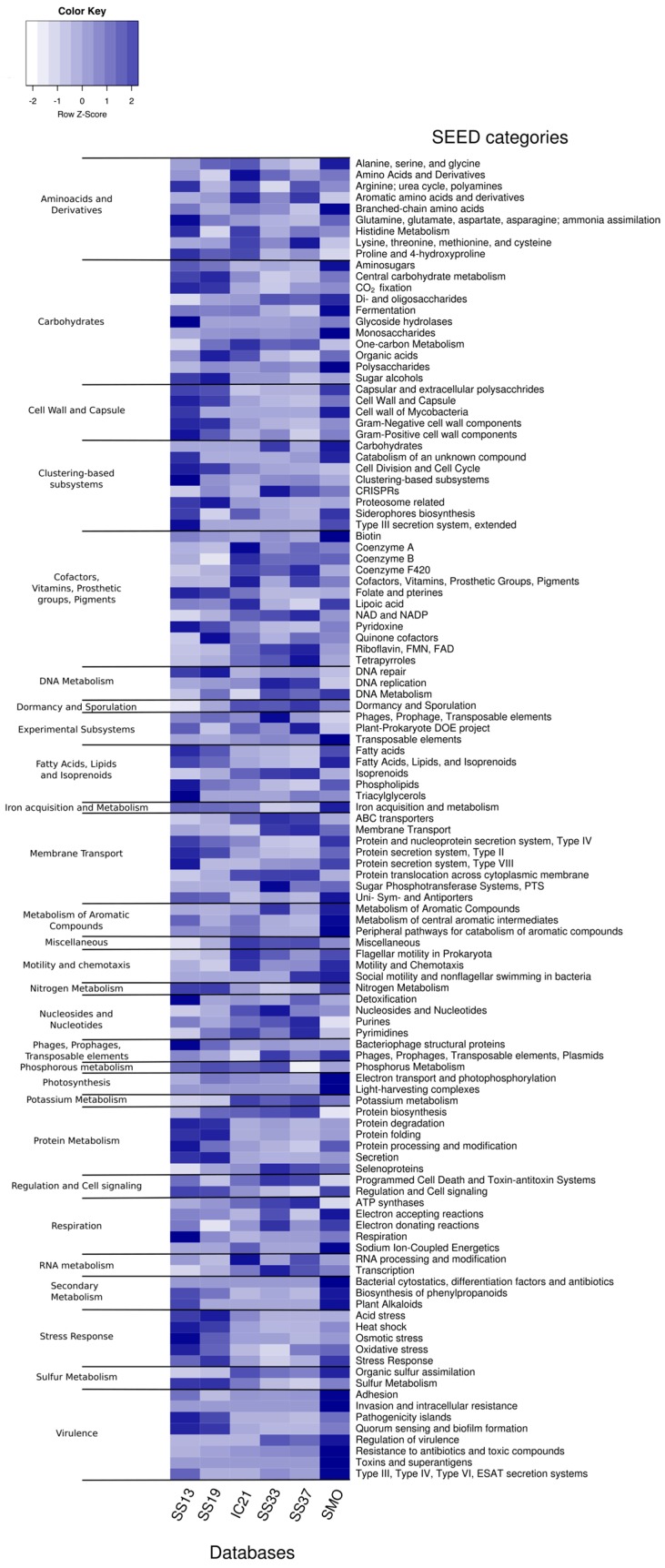
Heatmap depicting the proportion of proteins related to SEED subsystems in the salterns and soil databases. Salterns databases used are described in Table 1. SMO refers to the contigs resulting from the co-assembly of the two saline soil databases, SMO1 and SMO2. Scale has been applied by row.

Figure 4 shows the proportion of SEED subsystems [94] annotated in contigs from the saline soil databases compared to those of previously studied salterns databases. Most of the genes related to fermentation, monosaccharides and polysaccharides, membrane transport, iron acquisition, motility and chemotaxis were overrepresented in soil databases. Given that nutrient concentrations in soil habitats are usually very low [95], it is likely that the ability to move, communicate, and rapidly acquire nutrients when available may be advantageous for microorganisms thriving in them [96,97,98]. Soil environments are also more dynamic and heterogeneous than aquatic systems, especially in relation to water and nutrient content [95]. Thus, metabolically versatile microbes, able to use different carbon and energy sources may be able to survive to the frequent changes of those factors in soils. The high proportion of polysaccharides may also be caused by a large production of exopolysaccharides (EPS), which is related to the adhesion to solid surfaces such as soil particles, as well as with protection against stressors such as desiccation, high metal concentrations and other toxic substances [99] frequent in these environments. 

We found that genes classified into the category of pathogenicity, virulence and secondary metabolites were increased in soil databases versus salterns. It has been proposed that nutrient scarcity promotes the synthesis of antibiotics and thus, their resistance mechanisms too [100], which may explain our results. Also, soils are recognized for their large proportion of secondary metabolites-producers and thereby usually investigated in the search of new molecules for a wide range of uses and biotechnological applications [101]. It is worth noting that there are no known pathogens within haloarchaea and thus, databases with more bacteria could be expected to harbor more virulence genes. However, despite SS13 (13% salts) and SS19 (19% salts) possessing a bigger bacterial community, contigs obtained from soil databases contained the highest proportion of virulence and pathogenicity-related genes, highlighting the harsh nutritional conditions found in soils.

Photosynthesis-related genes were also overrepresented in soil databases, which contained a larger community of photosynthetic primary producers (Figure 3). Cell wall and capsule-related genes are in the range of the less saline SS13 and SS19 databases from saltern ponds, which comprise a higher proportion of bacterial representatives. 

The high proportion of sequences attributed to transposable elements in soil databases may indicate that in an oligotrophic and variable environment such as soil, genomic plasticity and acquisition of new traits may greatly contribute to fitness and survival. Strikingly, a higher proportion of reads devoted to the SEED subsystem for dormancy and sporulation was found in intermediate and high salinity saltern ponds, as compared to soil databases. Although it could be argued that soil microorganisms experience more frequent and unfavorable changes in their surrounding conditions and thus, may potentially be able to reduce and resume growth in a greater proportion than aquatic microorganisms, our data do not support this hypothesis. On the other hand, it has been proposed that the competition for resources, especially in extreme conditions, exerts pressure on activity versus dormancy [102], which would explain our results.

Although the SEED category corresponding to stress response may be hypothesized to be one of the most abundant in these environments, our results do not show that trend. Not all pathways and genes involved in the main mechanisms of osmoadaptation are included in that subsystem (e.g., trehalose biosynthesis, ions transport), which could partly explain this situation. Thus, we individually investigated the metabolic pathways related to osmoadaptation. Trehalose biosynthesis and betaine synthesis from choline and uptake from the environment seemed to be the prevailing compatible solutes accumulation mechanisms used by microbiota thriving in the studied soils (Table S2). In the aquatic databases, betaine was preferred over trehalose as osmolyte. Glycine betaine, ectoine and its derivatives as well as trehalose are among the most prominent osmolytes used by prokaryotes [103]. In our study, genes related to ectoine synthesis and transport were the least abundant of the analyzed osmolytes. Once determined as the major osmolyte in the extensive taxa Proteobacteria and Firmicutes as well as other bacteria [104], molecular approaches investigating osmoadaptation strategies in hypersaline environments have shown that the relative importance of ectoine as compatible solute in those environments is limited [22,23,56]. 

As in marine environments, light serves as a source of energy for heterotrophic communities living in aquatic hypersaline environments [4], as reflected in the number of reads affiliated to rhodopsins in salterns databases (Table S2). The prevalence and importance of rhodopsin genes in terrestrial environments are not well-known. In our study, the proportion of reads related to bacterial proteorhodopsins and haloarchaeal bacteriorhodopsins in saline soil databases were slightly lower than those of salterns databases. Nevertheless, despite the opacity of the soil matrix, our results showed that microorganisms thriving in these saline soils might retain their capacity for photoheterotrophy. 

### 3.6. Insights into Untapped Genomic Diversity through Metagenomic Binning

Binning techniques allow the grouping of sequences related to a specific taxon. Although it has been shown to be especially difficult to assemble environmental genomes from soil metagenomic databases due to high microbial diversity and usually insufficient sequencing coverage [105], the use of sequence composition and coverage variation data of taxa in the two studied metagenomes allowed us to recover four high quality MAGs (also denominated bins) from the soil databases (Table S4). 

#### 3.6.1. Estimation of the Phylogenetic Affiliation of Assembled MAGs

**Figure 5:**
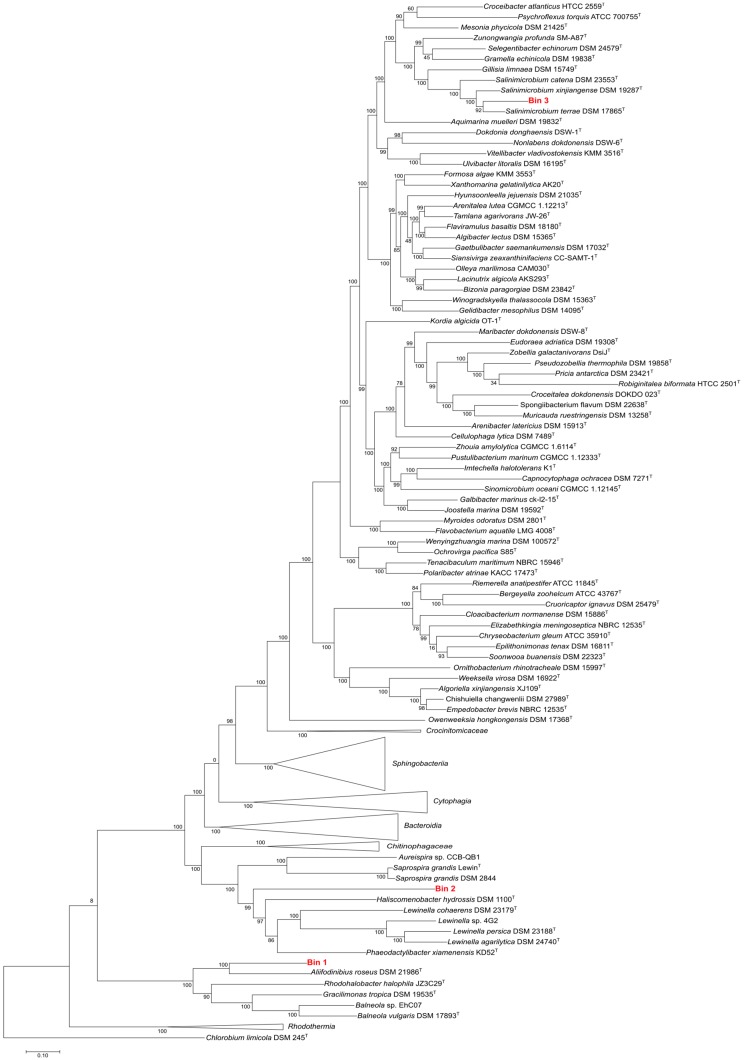
Genome phylogeny of the phylum Bacteroidetes using PhyloSift v1.0.1 marker genes [52]. The branch length units are substitutions per site and node labels are clade confidence estimates. Bins assembled in this study are marked in red. *Chlorobium limicola* DSM 245^T^ was used as outgroup. See Materials and Methods sections for further methodological details.

Three MAGs were classified as members of the domain Bacteria, while the less complete corresponded to an haloarchaeon (bin 4). Phylogenetic analysis revealed that the highest quality genome was part of the recently proposed Balneaolaeota phylum (bin 1), while the other two were related to Saprospirales (bin 2) and Salinimicrobium (bin 3) within the phylum Bacteroidetes (Figure 5). Further classification of the bin ascribed to Balneolaeota was attempted by average nucleotide identity (ANI) computation. ANI results indicated that it was not closely related to any of the Balneolaeota genera with available sequenced genomes (Table S3). However, the genus *Fodinibius* within Balneolaeota, which encompasses halophilic representatives, does not include any representative with sequenced genome and thus, the reconstructed bin may either belong to a member of the cited genus or a Balneolaeota-related yet undescribed new taxon. At this point, it is useful to keep in mind that environmental genomes mined from metagenomes usually represent “population” genomes, and no single species [25].

#### 3.6.2. Genomic Analysis of Assembled MAGs

Genomic analysis of the extracted MAGs revealed many proteins related to resistance to metals such as copper, cobalt, zinc, cadmium, mercuric, and arsenic as compared to their closest relatives with sequenced genomes. Thus, although the concentration of metals in the studied saline soils do not reach the legally agreed levels for considering them contaminated, prokaryotes in these habitats have needed special adaptations to cope with those amounts of cellular toxic compounds. 

In comparison with the rest of representatives of Balneolaeota with available genome at the time of the analysis, the related bin possessed a higher proportion of genes involved in DNA repair, dormancy and sporulation, glycine metabolism and resistance to toxic compounds. Also, it coded for carotenoid-synthesis genes and a proteorhodopsin, whose best BLAST hits corresponded to *Salinibacter* xanthorhodopsins. We did not detect any sequence related to these photochemically active membrane proteins in other Balneolaeota genomes, but Vavourakis et al. [25] reported the draft genome of an uncultured *Balneola* representative which encoded a putative sodium pumping rhodopsin. Therefore, Balneolaeota may harbor members with the ability to obtain energy from light. The already described taxa of the phylum Balneolaeota require 5–10% NaCl to grow and have been isolated form diverse habitats ranging from marine habitats to saline sediments. The main compatible solutes used by the members of this phylum seems to be diverse: while the *Balneola* available genome includes betaine and glycerol transporters, *Rhodohalobacter* type species genome codes for choline uptake proteins and a choline dehydrogenase and *Aliifodinibius roseus* DSM 21986 has no osmolyte-related gene annotated in its genome. Both betaine and choline transporters were annotated in MAG 1 genome, which is also the case for *Gracilimonas tropica* DSM 19535.

Saprospirales-related MAG had the most reduced pool of sequences dedicated to resistance to toxic compounds of the assembled bins. Genes related to capsule formation and dormancy state were identified in its genome. Aerobic (by means of cytochrome c oxidase) and anaerobic growth (by fermentation to butyrate) can be predicted from the draft genome of MAG 2.

The genome of the representative of *Salinimicrobium* recovered in our study encoded a choline uptake protein and the trehalose biosynthetic pathway. By their genomic sequence analysis, *Salinimicrobium xinjiangense* and *Salinimicrobium terrae* may also be able to use trehalose as compatible solute. The osmoadaptation strategy of the rest of the taxa from this genus, which comprise halotolerant to slightly halophilic strains, remains unknown. No sequence related to motility was detected. Anaerobic growth may be feasible as denitrification and fermentation-related proteins were annotated in the studied genome.

As for the haloarchaeal bin, ~22% of the annotated reads in SEED categories corresponded to membrane transport (mainly oligopeptides and branched amino acids transporters) and another ~13% were devoted to resistance to toxic compounds. Glycine betaine transporter OpuD and BetT choline uptake protein were also present, as was a choline sulfatase (EC 3.1.6.6). The presence of these transporters does not necessarily indicate utilization of salt-out strategy by this haloarchaeon, as these osmolytes may serve as carbon or nitrogen source, as well as thermoprotectants [106]. Although no rhodopsin gene could be detected in the partial genome, a bacterio-opsin activator domain-containing protein was annotated in it.

#### 3.6.3. Abundance of Balneolaeota-Related Bin in Hypersaline Environments

**Figure 6:**
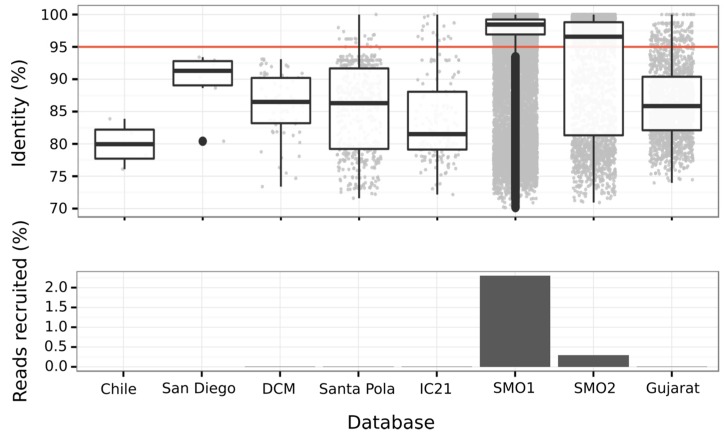
Percentage of metagenomic reads from diverse hypersaline environments recruited and their identity to the genomic sequence of bin 1. Saltern databases used were: Chile, corresponding to a crystallizer pond with 34% salinity from Cáhuil Lagoon (Chile) [58]; San Diego, resulting from the combination of the datasets obtained from Guerrero Negro solar saltern (Baja California Sur, Mexico) [24]; Santa Pola, as the combination of the metagenomes obtained from Santa Pola saltern (Alicante, Spain) [22,23] and IC21, corresponding to the metagenomic database of a concentrator pond with 21% salinity from Isla Cristina saltern (Isla Cristina, Huelva, Spain) [56]. DCM corresponds to a Mediterranean marine sample with a total salt concentration of 3.5% [57]. Saline soils are represented by the Odiel saltmarshes databases (SMO1 and SMO2) and the metagenomic datasets from soils located in the Great Rann of Kutch (Gujarat, India), combined in Gujarat [19].

Recruitment analysis showed that the Balneolaeota representative from which we could recover the genome was not abundant in hypersaline aquatic datasets from around the world (Figure 6), reinforcing the idea that some microorganisms may be highly adapted to either terrestrial or aquatic habitats. Our results are similar to those of Hollister et al. [18] in which a soil-specific group of organisms related to the genus *Balneola* was detected when studying a salinity range comprising soils and sediments. Recent genome-resolved metagenomic studies from hypersaline soda lakes also found that *Balneola*-related bin was not among the most abundant taxa thriving in these habitats [25]. Also, this binned genome was more abundant in SMO1 sample than in SMO2. A time series study would be needed to establish the relationships of the corresponding organism with the changing environmental conditions.

## 4. Conclusions

The dominant prokaryotic groups dwelling in the studied hypersaline soils (Halobacteria, Balneolaeota, Bacteroidetes, Rhodothermaeota and Nanohaloarchaeota) comprise well-known halophiles also reported in previous studies of other aquatic and terrestrial habitats. Other minor phyla with different tolerances to salinity were also present, probably due to micro-niches of soil harboring diverse environmental conditions supporting their survival and/or growth. Microbial diversity of the studied saline soils proved to be in the range of intermediate salinities saltern ponds analyzed for comparison. We suggest that there is a preference of some taxa for either aquatic (e.g., *Haloquadratum*) or terrestrial (e.g., Balneolaeota-related bin) habitats, while other members of hypersaline habitats may be similarly adapted to both conditions. Importantly, we reported the presence of Nanohaloarchaeota in soils and were able to assemble MAGs from microbial dark matter of saline soils for the first time. The metabolic potential of the microbiota thriving in these saline soils reflects its halophilic and terrestrial nature. We have highlighted the similarities and differences between the widely studied aquatic hypersaline habitats and the disregarded saline soil systems. While there are still major challenges in saline soils microbial ecology that need to be addressed to keep pace with its aquatic counterparts, this study constitutes an important contribution to the current knowledge of their prokaryotic phylogenetic and metabolic diversity. 

## Figures and Tables

**Table 1:** Features of the different databases from hypersaline habitats analyzed in this study.

Database Name	Habitat	Salinity	pH	No. of Sequences	Avg. Read Length (bp)	Percent of Assembled Reads (%)	No. of Contigs >1 kb/N50 (bp)	Ref.
SMO1	Saline soil	24.0 dS/m	7.8	1,289,630	628	26.7	25,001/1857	[59]
SMO2	Saline soil	54.4 dS/m	8.9	839,941	629			
SS13	Saltern	13% NaCl	8.0	1,481,803	305	63.7	8193/2280	[23]
SS19	Saltern	19% NaCl	8.0	1,241,633	361	65.3	12,742/2429	[22]
SS33	Saltern	33% NaCl	7.0	963,381	367	59.0	6040/2069	[23]
SS37	Saltern	37% NaCl	7.1	736,936	417	67.0	6589/2336	[22]
IC21	Saltern	21% NaCl	7.5	1,191,373	397	69.2	9270/2793	[56]

**Table 2:** Genus-level taxonomic affiliation of 16S rRNA genes identified in the reads from the two saline soil samples, SMO1 and SMO2. Assigned sequences have an identity over 95% and a minimum length of 100 bp. The category “Others” includes taxa represented by less of 1% of the sequences. They are colored by taxonomic group as follow: Euryarchaeota—red, Bacteroidetes—green, Gammaproteobacteria—light blue, Alphaproteobacteria—orange, and Actinobacteria—purple, Rhodothermaeota—gray, Balneolaeota—dark blue.

SMO1 Database		SMO2 Database	
Haloarcula	12.7%	Salinimicrobium	9.2%
Halorubrum	10.3%	Salinigranum	9.0%
Fodinibius	9.0%	Halolamina	8.7%
Halolamina	7.4%	Haloarcula	8.4%
Salinigranum	7.1%	Unclassified Halobacteria	6.3%
Unclassified Halobacteria	6.7%	Halobellus	4.6%
Salinibacter	4.1%	Unclassified Gammaproteobacteria	4.1%
Gracilimonas	3.4%	Gracilimonas	3.0%
Halohasta	3.4%	Halorubrum	2.7%
Halapricum	2.7%	Halomicroarcula	2.2%
Halobellus	2.5%	Marinobacter	2.2%
Natronomonas	2.5%	Natronomonas	2.2%
Halonotius	2.1%	Pseudidiomarina	2.2%
Haloplanus	2.1%	Altererythrobacter	1.9%
Halorientalis	1.9%	Halomarina	1.9%
Halorubellus	1.8%	Haloplanus	1.9%
Halomicroarcula	1.6%	Halorientalis	1.9%
Salinimicrobium	1.6%	Fodinibius	1.6%
Unclassified Ectothiorhodospiraceae	1.2%	Halomonas	1.6%
Unclassified Gammaproteobacteria	1.2%	Nocardiopsis	1.6%
Halalkalicoccus	1.1%	Unclassified Flavobacteriaceae	1.4%
Halomonas	1.1%	Idiomarina	1.1%
Others	12.7%	Others	20.4%

## Supplementary Materials

The following are available online at http://www.mdpi.com/2073-4425/9/3/152/s1. Table S1: Physico-chemical characteristics of the two soil samples designated as SMO1 and SMO2, Table S2: Fraction of reads (%) devoted to specific osmoadaptation functions from the studied hypersaline soils and salterns databases, Table S3: ANI values (%) between genomes of the type species from the phylum Balneolaeota and bin 1, Table S4: Completion and contamination of the retrieved bins, according to CheckM.
