# Supplementary material for: Metagenomic Insights into the Phylogenetic and Metabolic Diversity of the Prokaryotic Community Dwelling in Hypersaline Soils from the Odiel Saltmarshes (SW Spain)
Source: Genes (Basel). 2018 Mar 8;9(3):152. doi: 10.3390/genes9030152 (PMC5867873; doi:10.3390/genes9030152)
Supplement: Supplementary file 1 [file genes-09-00152-s001.pdf]

Table S1. Physico-chemical characteristics of the two saline soil samples designated as SMO1 and SMO2. For methodological aspects see Materials and Methods section.

Parameter	Units	SMO1	SMO2
$pH_{1:2.5}$	-	7.8	8.9
$EC_{1:5}$	dS/m	24.0	54.5
Salinity (1:5)	g/l	16.3	54.7
Water content	%	14.7	6.9
Organic matter	mg/kg	2,590	2,342
Organic carbon	mg/kg	N.D.	13,590
Total carbon	mg/kg	N.D.	17,640
Total nitrogen	mg/kg	1,070	890
Organic nitrogen	mg/kg	N.D.	780
Ammonia – water soluble	mg/kg	N.D.	3.03
Nitrite – water soluble	mg/kg	N.D.	0.04
Nitrate – water soluble	mg/kg	N.D.	257.0
Sulfate – water soluble	%	1.35	6.68
Phosphorus – water soluble	%	3.095	22.759
Chloride – water soluble	%	0.930	14.155
Sodium – water soluble	%	2.95	8.6
Magnesium – water soluble	%	0.13	0.88
Calcium – water soluble	%	0.27	1.32
Potassium – water soluble	%	0.56	0.12
Total copper	mg/kg	N.D.	74.1
Total zinc	mg/kg	N.D.	84.4
Total aluminium	mg/kg	N.D.	5.2
Total arsenic	mg/kg	N.D.	11.5
Total cadmium	mg/kg	N.D.	0.1
Total mercurium	mg/kg	N.D.	B.D.L.
Total lead	mg/kg	N.D.	16.8

N.D. - Not determined; B.D.L. - Below detection limit

Table S2. Fraction of reads (%) devoted to specific osmoadaptation functions and rhodopsins from the studied hypersaline soils and salterns databases. Salterns databases used are described in Table 1. SMO refers to the contigs resulting from the co-assembly of the two saline soil databases, SMO1 and SMO2.

	**SS13**	**SS19**	**IC21**	**SS33**	**SS37**	**SMO**
Betaine biosynthesis from glycine	0.0265	0.0356	0.0180	0.0288	0.0053	0.0049
Betaine biosynthesis from choline	0.5531	0.2161	0.0717	0.0222	0.0101	0.1609
Betaine uptake	0.3531	0.4003	0.1741	0.0549	0.0281	0.0748
Choline uptake	0.2671	0.2298	0.1181	0.1303	0.0943	0.0967
Ectoine uptake	0.0022	0.0008	0	0	0.0006	0.0014
Ectoine biosynthesis	0.1338	0.0502	0	0	0	0.0254
Trehalose biosynthesis	0.1285	0.2238	0.0738	0.1027	0	0.2893
Trehalose uptake	0	0	0.0004	0	0	0.0049
Proteorhodopsin	0.0442	0.0506	0.0144	0.0040	0.0018	0.0071
Bacteriorhodopsin	0.0396	0.0387	0.0570	0.0830	0.0838	0.0233

Table S3. ANI values (%) between genomes of the type species from the phylum Balneolaeota and bin 1.

	***Aliifodinibius roseus* DSM 21986^T**	***Balneola vulgaris* DSM 17893^T**	**Bin 1**	***Gracilimonas tropica* DSM 19535^T**	***Rhodohalobacter halophilus* JZ3C29^T**
***Aliifodinibius roseus* DSM 21986^T**	100.0	66.6	69.8	67.9	67.6
***Balneola vulgaris* DSM 17893^T**	66.6	100.0	66.3	68.9	67.4
**Bin 1**	69.8	66.3	100.0	67.8	67.4
***Gracilimonas tropica* DSM 19535^T**	67.9	68.9	67.8	100.0	68.3
***Rhodohalobacter halophilus* JZ3C29^T**	67.6	67.4	67.4	68.3	100.0

Table S4. Completion and contamination of the retrieved bins, according to CheckM v1.0.5.

Bin Id	Marker lineage	# genomes	# markers	# marker sets	0	1	2	3	4	5+	Completeness	Contamination	Strain heterogeneity
Bin 1	k__Bacteria (UID2570)	433	274	183	33	235	6	0	0	0	86.65	2.24	0.00
Bin 2	p__Bacteroidetes (UID2591)	365	298	198	142	152	3	1	0	0	53.37	2.02	0.00
Bin 3	k__Bacteria (UID203)	5449	104	58	63	40	1	0	0	0	47.15	1.72	0.00
Bin 4	k__Archaea (UID2)	207	149	107	133	16	0	0	0	0	10.11	0.00	0.00
